# Foreign Summary

**Published:** 1839-08-03

**Authors:** 


### FOREIGN SUMMARY.

We publish the autopsy of Lady Flora Hastings, who unfortunately obtained a most unenviable and undeserved notoriety. She obviously laboured under a tuberculous peritonitis. This disease is little understood in this country and in England. It is extremely slow in its progress, begins as a chronic affection, the abdomen gradually enlarges with an obscure pain, becoming acute only in a small proportion of cases. At the same time there is occasional vomiting, difficult digestion, and often obstinate constipation. These may very readily be mistaken for pregnancy, and thus give rise to a serious error.

It is stated that there was some unorganized yellow matter in the peritoneal adhesions. This was the tubercular deposit, which is not expressly described, either from the case being merely intended for the eye of the public, or from a desire to conceal the original cause of the complaint.

### LADY FLORA HASTINGS.

This lady has been buried in Scotland. Before she died, she gave positive orders to have her body opened after her death.

"*Appearances observed on inspecting the body of the late Flora Hastings, July 5th*, 1839.

"There was great emaciation of the whole person.

"In the chest—the heart and lungs were in a perfectly healthy state; but there were extensive adhesions of the pleura (or membrane) covering the right lung to that which lines the ribs—evidently of long standing.

"In the abdomen there were universal adhesions of the peritoneum (or membrane which lines the cavity and covers the viscera,) so that it could not be said that there was a single organ which was not at every point on its surface, intimately connected with the parts in its vicinity. The liver was very much enlarged, extending downwards as low as the pelvis, and upwards, so as very materially to diminish the capacity of the right cavity of the chest.—The gall bladder contained a small quantity of bile.

"The liver was of a very pale colour, but its structure was not materially different from what exists in the healthy state. The stomach and intestines were distended with air; their coats, especially the muscular, were very much attenuated. The spleen and pancreas were free from disease. Some of the mesenteric glands were enlarged. There were a few small deposites of unorganized yellow matter, apparently in the substance of the adhesions.

"The uterus and its appendages presented the usual appearances of the healthy virgin state.

"From the character of the adhesions it was plain that they could be referred only to inflammation at some former and distant period of time. The effect of them must have been to interrupt the passage of the contents of the stomach and intestines, and in various ways to interfere with the due performance of their functions.

W. F. Chambers, M.D.
Henry Holland, M.D.
Astley Cooper,
B. C. Brodie,
John Merryman."

*From a Lecture of Dr. Magendie, on the Pathological Effects of Defibrinising the Blood.*

This extract we give, after having recently witnessed a severe case of dysentery, in which ophthalmia and abscess of the cornea took place at the close of the disease, almost without pain. In dysentery, the secretions are decidedly alkaline, and in severe cases of the disease the bluish and purple colour of the extremities shows that the blood has in a great degree lost its coagulability. When drawn from a vein the change is still more obvious. To a certain extent, this would bear out the ideas of Dr. Magendie.

The animal before us was bled before the lecture, in order that we might ascertain the condition of his blood. You see that scarcely any coagulum has formed; in the midst of the serum floats a semi-liquid mass of pseudo-fibrine. I had nearly forgot to point out an interesting appearance, which we also observed in the preceding dog, I mean an eruption resembling forcibly the petechiæ of typhus. There are small red points to be seen, wherever the hair is thinly scattered; several of them, indeed, cover a tolerably large extent of surface. Judging from the colour of these spots we are authorised in considering them to be constituted by blood extravasated between the layers of the skin, or into the subcutaneous cellular membrane; in fact, nothing more or less than ecchymoses. It is perfectly clear that the modifications occurring in the composition of the fluid are the cause of the symptoms under which the animal suffers, because previously to the performance of the experiment he was in good health. But it is not so clear whether the loss of one of its elements, or its having ceased to be coagulable, or those two altered conditions combined, have caused its extravasation.

Theoretically speaking, either supposition is equally tenable; but no difficulty can be felt in coming to a formal decision, if we find that the same morbid phenomena are producible whilst the blood still retains its normal composition. If such be the case, no doubt can possibly be entertained respecting the influence of deficient coagulability; and, in truth, the experiments we had previously made on the introduction of the subcarbonate of soda in solution into the veins were, from their result, sufficient to put an end to our uncertainty. We knew that the fluid had the power of effectually opposing the formation of the coagulum, but also that it possessed no direct poisonous influence. Veterinary surgeons are in the constant habit of injecting purgatives and other energetic substances into the vascular system of horses, and yet those animals suffer no other symptoms than those which follow the ingestion of the same medicines into the stomach. But the case is widely different when a solution of subcarbonate of soda is the injected material; if you desire to be convinced of this, look at the animal I now bring forward. Eight days past, a small quantity of that salt—ten grammes as nearly as possible—were introduced into his veins, and he, in consequence, fell extremely ill. The heart, lungs, stomach, all the viscera, in short, were simultaneously affected; exhalation of blood took place on all the surfaces, and in the substance of the various parenchymatous structures, as though that fluid had lost all its fibrine. Nevertheless, no change had occurred in its constitution; the whole original amount of fibrine still remained in the circulation. Why, then, these extravasations? you will ask; because the liquid had been deprived of the most important of its properties, coagulability. The symptoms observed in this dog manifested a still more dangerous character than those occurring in defibrinised animals, because in it the blood did not, as in the latter, contain a sort of imperfect substitute for the solidifiable principle (pseudo-fibrine.) The animal was beginning to recover its strength, and was, in fact, convalescent two days ago; but I then repeated the injection of ten grammes of subcarbonate of soda, and all the former symptoms immediately disappeared. The eyes which had only been rendered very red and weeping by the first experiment, now present very serious lesions. The cornea has lost its transparency, and is ulcerated superficially in several spots; the alimentary canal is the seat of disorders of the kind attending every general morbid state caused by diminished coagulability of the blood; the stools are watery, and of a reddish-brown colour, as if serum and colouring matter had been thrown out on the surface of the bowels; the functions of the stomach are at a stand still; there is absolute inappetence; the pulmonary tissue is also most seriously affected. On applying my ear to the thorax I detect various ronchi, caused by the extravasation of blood into the bronchi and vesicles, and between the capillary tubes.

I opened the jugular, in order to learn whether the blood be susceptible of clotting: the clot, we find is small and friable; in fact, in a condition perfectly harmonizing with the symptoms we witness. It is too much altered in constitution to admit of a normal exercise of the functions: it is not sufficiently so to put an end to their play. Hence, whether the fibrine remain in the vessels or not, the effects of non-coagulability are exactly the same. This is an important point, for it shows that when we find the blood of patients affected with fever, to give, on analysis, all its normal elements in their normal proportion, we are not at once entitled to conclude that that blood is fit for circulation. Before we pronounce such an opinion, we must also examine the clot; if it be soft and friable, our patient is in a most dangerous condition; if it be completely absent, death is almost inevitable. Such is the degree of precision of which these experimental studies are capable, that we might boldly state this problem :—A given quantity of fibrine being removed, or of subcarbonate of soda being injected into the veins, what will be the nature of the symptoms? There is not one among you, gentlemen, who would not, with the knowledge he now possesses, be capable of resolving the problem. It is possible, too, that the simple inspection of the clot, when performed by persons thoroughly acquainted with the history of the blood, would suffice to point out, in cases of illness, what organs are affected, and what is the nature of their lesions; the verification of this hint I leave as an interesting subject for your labours.—*Lancet.*

---

*Phlegmonous Abscess of the Lung in Process of Cure.*—At a meeting of the Pathological Society, Dr. Corrigan presented an example of phlegmonous abscess of the lung, taken from a patient who suffered from pleuro-pneumonia seven weeks previous to his death; he recovered in a great measure from this attack, but a slight hæmoptysis continued to recur daily; an excess in regimen was followed by a severe hæmorrhage, which proved rapidly fatal; the lungs were healthy, with the exception of the left lung, where an abscess of about the size of a walnut, lined with coagulable lymph, existed; it communicated with a large bronchial tube; this abscess, and the neighbouring bronchial tube, were found filled with some coagulated blood; there were no traces of tubercle in any portion of the lung.

*Disease of the Mitral Valve, without valvular Murmur.*—Dr. Stokes presented a specimen of disease of the mitral valve. It was principally remarkable on account of the physical phenomena observed during life. In this case the patient had long laboured under symptoms of morbus cordis, with chronic bronchitis; and when the first was seen by Dr. Stokes, had a distinct and rough bruit de soufflet with the first sound of the heart. The second sound was unaffected. He remained under observation for several months, and always presented the same phenomena. In eighteen months he again came under Dr. Stokes' care, in a very advanced stage of dropsical disease. All bruit de soufflet had disappeared, and the heart merely presented the usual signs of hypertrophy. He died in about a month, during which time he was repeatedly examined, but no valvular murmur was ever detected; the heart was much enlarged; the left auricle greatly distended and thickened; the auriculo-ventricular valve was greatly diseased, and the orifice presented the semilunar contraction described by Mr. Adams; the opening was not more than two lines in breadth; numerous irregular deposits of earthy matter existed on the ventricular side of the valve. Dr. Stokes detailed the particulars of another case, in which a remarkable auricular contraction of the mitral valve had been found, in which no bruit de soufflet whatever had existed for a considerable time previous to death.—*Dublin Journal.*

---

*On the Use of Camphor in Certain Affections of the Respiratory Apparatus.* By F. V. Raspail.—The following is the substance of a letter which M. Raspail has recently addressed to several of the French medical journals. It is seldom that we pay any attention to the proposal of remedial agents by non-medical men; but the distinguished character of M. Raspail, both as a chemist and an observer of nature, entitle any remarks which fall from his pen to more than ordinary attention.—*London Lancet.*

"The substance which M. Raspail recommends to the notice of medical practitioners, is camphor. It may be used in two forms: a piece of camphor is placed in a small tube of straw, or in a small quill, and this is formed into a little cigar, which the patient may smoke, not in a state of ignition, but cold, by simply inhaling the air through it. The saliva excited by inspiring the camphor, should be swallowed. The second form consists in the application of a piece of lint, moistened with a saturated alcoholic solution of camphor, and covered with a piece of oiled silk, caoutchouc, or any other impermeable substance, to the affected part.

"M. Raspail assures us, as the result of considerable experience, that in all cases of respiratory affections, such as those popularly denominated cold, catarrh, influenza, &c., the constant use of the camphor cigar and lotion will produce speedy amendment, and when the lungs are merely congested, almost instantaneous relief. He has also seen some cases which lead him to believe that the constant use of camphor, in the way just mentioned, is capable of dissipating the incipient symptoms of pulmonary consumption. The pain occasioned by adherence of the two pleuræ, popularly known by the term 'stitch in the side,' M. Raspail has seen dissipated in a wonderfully short space of time, by the application of the camphorated compress, and the use of the cigar. There are several other affections and diseases in which M. Raspail thinks that camphor might be employed with great advantage; but we think it sufficient, for the present, to direct attention more particularly to those of the respiratory apparatus. At the conclusion of his letter, the author assures us that his communication has been made solely from a desire of benefiting his fellow men, and with a hope that medical practitioners will repeat his experiments on an extensive scale, the more especially as the remedy, unlike so many others, can do no harm, if it effect no good."—*French Lancet,* Nov. 17, 1838.

